# Comparison Between Nr4a Transcription Factor Regulation and Function in Lymphoid and Tumor Treg Cells

**DOI:** 10.3389/fimmu.2022.866339

**Published:** 2022-04-19

**Authors:** Takashi Sekiya

**Affiliations:** ^1^ Section of Immune Response Modification, Department of Immune Regulation, The Research Center for Hepatitis and Immunology, National Center for Global Health and Medicine, Ichikawa, Japan; ^2^ Department of Immune Regulation, The Research Center for Hepatitis and Immunology, National Center for Global Health and Medicine, Ichikawa, Japan

**Keywords:** Treg, antitumor immunity, autoimmunity, Nr4a, PGE2

## Abstract

Although the “lymphoid” function of regulatory T (Treg) cells is crucial for organismal homeostasis, these cells are also known to suppress the antitumor immune response in the tumor microenvironments. Thus, a detailed understanding of Treg cell maintenance and function in both lymphoid organs and tumor environments may help to establish novel methods for the reactivating antitumor immunity, while retaining necessary immune tolerance towards self and non-hazardous antigens. Previous studies have hypothesized that Treg cells behave similarly in lymphoid organs and in tumor environments; however, few studies have been conducted specifically researching Treg cell activity in tumor environments. In addition, several recent studies identified a novel mechanism regulating Treg cell function in tumor environments. Our group has previously described the critical roles of the Nr4a family of nuclear orphan receptors, comprising Nr4a1, Nr4a2, and Nr4a3, in the differentiation and maintenance of Treg cells in lymphoid organs. Subsequently, it was found that Nr4a factors help to maintain Treg cell function in tumor environments, thereby playing a suppressive role against T cell antitumor immunity. Importantly, there were some differences between the activities of these Nr4a factors under these conditions, including the specific function of the COX/PGE2 axis in tumor environments. This review was designed to investigate the role of Nr4a factors in the regulation of Treg cell activities both in the lymphoid organs and tumor environments, highlighting the commonalities and differences in their behaviors between Treg cells in these two different environments.

## Introduction

Regulatory T (Treg) cells are a central mediator of immune homeostasis, functioning to suppress unwanted immune reactions against self- and commensal-antigens (1). Various molecules have been identified as factors in the generation of effective immunosuppressive Treg subsets. In addition to the more “common” factors, including lineage-specifying transcription factor Foxp3 and inhibitory receptor CTLA-4, several other molecules have been identified to be uniquely expressed in specific Treg subsets and confer unique functions on these groups of cells. Recent studies have found that tissue-specific transcription factors endow Treg cells with unique functions within their corresponding tissue environments (2). For example, Treg cells that express PPAR-γ, the central transcription factor in adipocyte differentiation, reside in visceral adipose tissue and restore insulin sensitivity in obese mice in the presence of PPAR-γ agonists (3). It is also important to note that Treg cell phenotypes are also known to change in response to the tumor microenvironments (TME), contributing to accelerated tumor growth *via* augmented suppressive activity against antitumor immunity (4). A detailed understanding of these environment-specific phenotypes has significant clinical value, as the systemic manipulation of Treg cell activities is expected to trigger immune-related adverse events (irAEs).

A series of recent studies completed by our group revealed that the “Nr4a family” of nuclear orphan receptors play critical roles in the differentiation and maintenance of Treg cells. Nr4a factors have the ability to induce Foxp3, and their deletion results in a lack of Treg cell differentiation and the induction of severe autoimmunity (5–7). Nr4a factors are also highly expressed in mature Treg cells, where they help to maintain Treg cell lineage stability *via* their activation of the Treg cell-associated genes and repression of the Th-effector genes (8). Collectively, Nr4a factors have been revealed to maintain immune homeostasis by shifting the Th/Treg balance toward Treg cells. Importantly, another recent study revealed that these Nr4a factors are also highly expressed in tumor Treg cells, augmenting their suppressive activity on antitumor immunity (9).

Thus, Nr4a factors sustain Treg cell activity during both normal homeostasis and during the suppression of antitumor immunity. Although Nr4a factors are expected to behave similarly in Treg cells in both lymphoid organs and tumor tissues, several differences have also been observed. A deeper understanding of these differences are likely to facilitate the development of novel methods for the selective disruption of Treg cells in tumor tissues, while maintaining their function in lymphoid organs. This review will discuss both the unique and common molecular mechanisms governing the behaviors of Nr4a factors in lymphoid and tumor Treg cells. In addition, we will refer to Treg cells function in the maintenance of normal immune homeostasis “lymphoid Treg”, while Treg cells associated with antitumor immunity in the TME will be referred to as “tumor Treg”.

## The Nr4a Family of Nuclear Orphan Receptors

**Figure 1:**
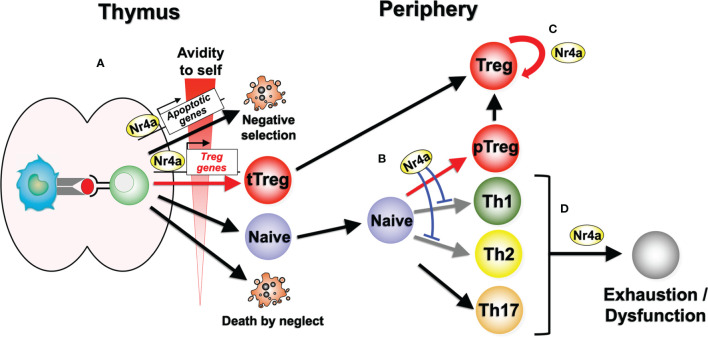
Nr4a factor functions in lymphoid Treg cell differentiation and maintenance. **(A)** Nr4a factors function as effectors in thymocyte fate decisions according to the avidity for self antigens. Strong avidity to self antigens induces higher amount of Nr4a factors which promotes apoptosis or Treg cell differentiation. “Death by neglect” represents the event of cell death that happen to thymocytes that could not recognize peptide-loaded self-MHC molecules during positive selection. **(B)** In the periphery, Nr4a factors promote Treg cell differentiation from naive T cells, while repressing Th1 and Th2 cell differentiation. Regulation of Th17 cells by Nr4a factors are still in debate. **(C)** Nr4a factors maintain the lineage stability of Treg cells. **(D)** Nr4a factors promote effector T cell exhaustion and/or dysfunction.

The Nr4a family of transcription factors Nr4a1, Nr4a2, and Nr4a3 belong to the nuclear receptor superfamily, and share a common structure, including an AF-1 transactivation domain in the N-terminus, a highly conserved DNA-binding domain, and a ligand-binding domain in the C-terminal portion of the protein (10–12). Nuclear receptors are known to be ligand-regulated; however, several lines of evidence suggest that the Nr4a factors are constitutively active in a ligand-independent manner. X-ray crystallography has revealed that the ligand-binding domain of Nr4a2 contains no cavity as a result of bulky hydrophobic residues in the region normally occupied by the ligands (13). Instead of regulation by ligand binding, Nr4a factors exhibit an intrinsic conformation similar to that of ligand-bound, transcriptionally active nuclear receptors (13). As a result, the transcriptional activity of Nr4a factors primarily depends on their expression levels, interaction partners, and their posttranslational modifications (14), although some molecules have been shown to additionally augment the basal activity of Nr4a factors (15, 16). The regulation of Nr4a factor expression in T cells is highly specific to the T cell receptor (TCR) signaling pathway, showing highly limited response to non-TCR stimuli such as cytokine signaling (17, 18). Indeed, Nr4a1-GFP mice, in which GFP expression is synchronized with *Nr4a1* expression, have been widely utilized as tools that specifically mark antigen-stimulated T cells (18). Nr4a factors induced by TCR stimulation exert various tolerogenic functions in T cells, including negative selection of self-reactive thymocytes (19–22), differentiation and maintenance of Treg cells (5–8), repression of Th-associated molecules (23), and the induction of hyporesponsive states (**Figures 1A, D**) (24, 25).

Nr4a factors primarily exert their function by transcriptionally regulate their target genes, as evidenced by the fact that mutant forms of Nr4a factors that lack either transcriptional activation domains or DNA binding domain, lack their ability to induce Treg cells (5), or even act as dominant negatives in the negative selection of self-reactive thymocytes (22). Increasing numbers of transcriptional regulators have been revealed as co-factors for Nr4a-mediated transcriptional events, including transcription factors, chromatin activators, and chromatin repressors (14). On the other hand, although Nr4a factors positively regulate pro-apoptotic gene expression, including FasL, TRAIL, NDG1, NDG2, and Bim (20, 26–28), their non-transcriptional activity has also been reported in the induction of apoptosis. Nr4a1 has been reported to translocate to mitochondria, whereby they convert anti-apoptotic Bcl-2 into pro-apoptotic mediator to accelerate the release of cytochrome *c*, promoting apoptosis of various types of cells including antigen-stimulated thymocytes, in transcription independent manner (29–33).

## Roles of Nr4a Factors in Treg Cell Differentiation

By screening Treg cell-enriched transcription factors, our group initially identified Nr4a factors as inducers of Foxp3 in conventional CD4^+^ T cells (5). Induction of Foxp3 by Nr4a factors is mediated by their function as transcription factors, as the mutant forms of Nr4a factors that lacked DNA binding ability or transactivating ability could not induce Foxp3 (5). Subsequent studies then showed that Nr4a factors play a crucial role in the development of thymic Treg (tTreg) cells. tTreg cells do not develop in mice in which all Nr4a factors are triple deleted (Nr4a-TKO), but single knockouts of each Nr4a factor produced normal tTreg cell populations, suggesting some degree of redundancy in their activity (6). By lacking tTreg cell development, Nr4a-TKO mice died soon after birth of severe autoimmunity (6).

Our recent study revealed that Nr4a factors play important roles in the differentiation of naive CD4^+^ T cells into Treg cells also in the periphery (pTreg cells) (23). By analyzing the differentiation of naive CD4^+^ T cells, Nr4a-TKO cells showed significantly attenuated Treg cell differentiation and accelerated Th1 and Th2 differentiation when compared with wild-type cells. As the result of the attenuated Treg cell differentiation and the accelerated Th2 differentiation, Nr4a-TKO naive CD4^+^ T cells are more allergic than wild type ones, inducing allergic airway inflammation in recipient lymphopenic mice. Furthermore, it was shown that the pharmacological activation of an engineered Nr4a molecule prevented allergic airway inflammation by shifting the differentiation of naive CD4^+^ T cells from Th2 to Treg cells (23).

As mentioned, Nr4a transcriptionally regulates Treg cell differentiation (5–8, 23). Nr4a factors positively regulate several, but not all, of Treg cell-associated genes during Treg cell differentiation (7, 23). In the induction of Treg cell-associated genes, although Nr4a factors by themselves can induce some of their target genes, other targets seem to be dependent on the simultaneous function of co-factors, including the Ets family of transcription factors (23). In addition, Nr4a factors not only function as transactivators, but also as repressors (7, 8, 23). Nr4a factors on their own can repress several Th2 and Tfh-associated genes, including their effector cytokines IL-4 and IL-21 (7, 8, 23). On the other hand, Nr4a factors also repress various Th2-associated genes indirectly, by suppressing the positive feedback loop of Batf factors, the key regulators for Th2 differentiation (23).

Collectively, Nr4a factors maintain immune homeostasis by promoting Treg cell differentiation both in the thymus and periphery, while suppressing Th cell differentiation, particularly that of Th2 cells (**Figures 1A, B**). In doing so, Nr4a factors promote expression of a part of Treg cell-associated genes, while repressing Th2-associated genes, on their own or in cooperation with other transcription factors.

## Roles of Nr4a Factors in the Maintenance of Lymphoid Treg Cells

Treg cells primarily develop from precursor thymocytes that experience strong TCR signaling *via* recognition of self-antigens with high avidity (34). Thus, developing Treg cells express high levels of Nr4a factors (7). Expression of all three Nr4a family member is maintained at high levels in Treg cells after their egress from the thymus, suggesting their continuous functions in peripheral mature Treg cells (18, 35–37). This assumption was recently evaluated in our group by establishing a mouse model in which all of the Nr4a family members were specifically deleted in mature Treg cells (Foxp3^Cre^-Nr4a-TKO mice) (8). Treg cells in these mice demonstrated a global reduction in Treg cell-associated gene expression, while showing upregulation of effector cytokine genes of Tfh and Th2 cells, including *Il4* and *Il21*. These Foxp3^Cre^-Nr4a-TKO mice also developed severe autoimmunity with elevated serum IgE and IgG1 levels, which are both associated with Th2-type immune reactions. Collectively, these data suggest that Nr4a factors are critical to the maintenance of lymphoid Treg cell integrity *via* their positive regulation of the Treg-associated genes and repression of Th2 and Tfh gene expression (**Figure 1C**).

## Roles of Nr4a Factors in the Maintenance of Tumor Treg Cells

**Figure 2:**
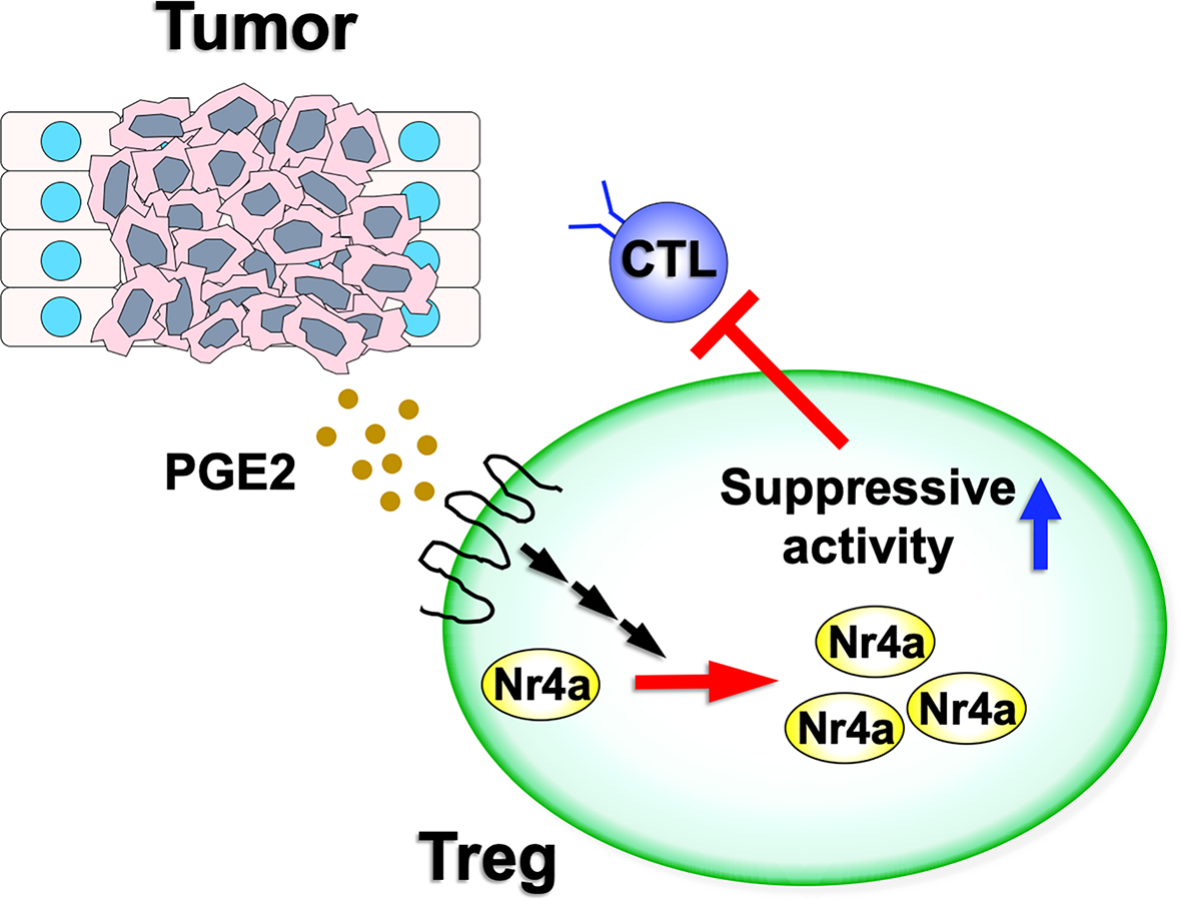
Nr4a factor functions in tumor Treg cells. Various tumor microenvironments are enriched for PGE2. As PGE2 has the ability to upregulate both Nr4a1 and Nr4a2, this increased PGE2 expression may augment Nr4a factor activity and increase the suppressive activity of the Treg cells on antitumor immunity.

Recently, Hibino et al. investigated the function of Nr4a factors in tumor Treg cells (**Figure 2**) (9). Their investigations revealed augmented antitumor immunity in mice in which both Nr4a1 and Nr4a2 were deleted specifically in Treg cells (Foxp3^Cre^Nr4a1^fl/fl^Nr4a2^fl/fl^ mice; referred to as “Foxp3^Cre^Nr4a-DcKO mice”), suggesting that the suppressive activity of tumor Treg cells was attenuated upon loss of Nr4a1 and Nr4a2. Accumulation of Treg cells in tumor-draining lymph nodes (TDLN) was also inhibited in Foxp3^Cre^Nr4a-DcKO mice, and the fractions of CD8^+^ T cells and proliferating CD8^+^ T cells were significantly increased in the TDLN of Foxp3^Cre^Nr4a-DcKO mice when compared to the wild-type. Furthermore, analysis of tumor tissues from the Foxp3^Cre^Nr4a-DcKO mice were also characterized by increased CD8^+^/Treg ratios and elevated CTL activity when compared to the wild-type mice. Investigation of the Treg cells from both the TDLN and tumor tissues revealed downregulation of Foxp3 and CTLA-4 in the Foxp3^Cre^Nr4a-DcKO mice when compared with the wild-type mice (9). Collectively, these data revealed that Nr4a factors, particularly Nr4a1 and Nr4a2, play important roles in the maintenance of tumor Treg cells.

## Pharmacological Inhibition of Nr4a Factors Attenuates Tumor Treg Cell Activity and Induces the Antitumor Immune Responses

In a subsequent study, Hibino et al. demonstrated that both camptothecin (CPT) and topotecan inhibit the transcriptional activity of Nr4a factors (9). Although CPT and topotecan have been known as topoisomerase inhibitors (38), other topoisomerase inhibitors did not demonstrate a similar effect, suggesting that the suppression of Nr4a factors by CPT and topotecan is independent of their inhibition of topoisomerase (9). CPT was found to suppress Treg cell differentiation while promoting the induction of IFNγ^+^ Th1 cells in naive CD4^+^ T cells (9). Furthermore, treatment of Treg cells with CPT reduced Foxp3 and other Treg-associated genes (9). However, these effects were not observed in Nr4a-DcKO Treg cells, indicating that CPT reduces Foxp3 through its direct inhibition of Nr4a (9).

Hibino et al. also investigated the effects of increased Nr4a factor expression on Treg cell characteristics. PGE2, an enzymatic product of COX-2, has been shown to induce Nr4a2 in intestinal tumors (39, 40). Given this, it was not surprising that treatment with PGE2 upregulated *Foxp3* and *Ikzf4*, both direct targets of Nr4a, in Treg cells (9). These effects were abolished in Nr4a-DcKO Treg cells, suggesting that PGE2 upregulated Treg cell-associated genes *via* induction of the Nr4a factors (**Figure 2**). In contrast, SC-236, a structural analog of the clinically useful COX-2 inhibitor celecoxib, reduced the expression of Nr4a factors as well as *Foxp3* and *Ikzf4* in tumor Treg cells.

Hibino et al. further examined the effects of these Nr4a inhibitors on tumor Treg cells and antitumor immunity. Both CPT-11, a less toxic prodrug of CPT, and SC-236 significantly reduced the growth of 3LL tumor cells, which constitutively express COX-2 and PGE2 (41), in a CD8^+^ T cell-dependent manner. The antitumor effects of CPT-11 and SC-236 were also shown to be further potentiated when used in combination (9). In TDLNs of mice treated with CPT-11and SC-236, it was found that Treg cell population was reduced, while the proliferating CD8^+^ T cell population was increased. Treg cells in the TDLNs of these mice exhibited attenuated expression of the Treg-signature genes, including *Foxp3* and *Ikzf4*, and attenuated suppression activity (9).

Altogether, pharmacological inhibition of Nr4a factors showed an efficient attenuation of tumor Treg cell activity and augmented the antitumor immune responses *in vivo*.

## Commonalities and Differences in the Nr4a Factor Mediated Regulation of Lymphoid Treg and Tumor Treg Cells

As described above, Nr4a factors play important roles in both lymphoid Treg and tumor Treg cell homeostasis and function. Although a detailed comparison has not been done yet, the behaviors of these Nr4a factors are hypothesized to be largely similar between these two cell populations. This is supported by the fact that Nr4a factors sustain Treg cell lineage, at least in part, by positively regulating Foxp3 in both lymphoid Treg and tumor Treg cells (8, 9). In addition, Nr4a family members exhibit a high degree of functional redundancy in both lymphoid Treg and tumor Treg cells, as demonstrated by the fact that none of the single knockouts showed any substantial defects in either Treg population (8, 9). Nr4a factors also negatively regulate Th-associated effector molecules in both lymphoid and tumor Treg cells (8, 9).

However, there are still some significant differences in the behaviors of Nr4a factors between lymphoid and tumor Treg cells. These include the fact that although Foxp3^Cre^Nr4a-DcKO mice do not develop autoimmunity under normal homeostatic conditions (8), they did demonstrate accelerated antitumor immunity (9). This observation suggests that the suppressive activity of tumor Treg cells on antitumor immunity is more sensitive to Nr4a availability than that of the lymphoid Treg cells on autoimmune response. Second, while the Th2- and Tfh-associated functions are upregulated in lymphoid Treg cells upon disruption of Nr4a function (8), deficiency of Nr4a factors in tumor Treg cells presents with increased activation of the Th1- and CTL-associated antitumor immune activity (9). This observation suggests that the function of Nr4a factors in Treg cells is influenced by their native environment. Although experimental validations are necessary, possible mechanisms for this include differences in the corroborating factors and/or post-translational modifications of Nr4a factors in response to differences in these microenvironments. Lymphoid and tumor Treg cells exist in vastly different microenvironments with each occurring in niches with significantly different metabolites and cytokines (42). Third, the expression of Nr4a factors in lymphoid and tumor Treg cells seem to be differently regulated. Although TCR signaling is undoubtedly the dominant positive regulator for Nr4a in both lymphoid and tumor Treg cells (17, 18), the COX-2/PGE2 pathway may enhance their expression in tumor Treg cells, especially since PGE2 is strongly enriched in various tumor microenvironments (43). In fact, Hibino et al. described increased Nr4a expression in Treg cells from a PGE2-positive 3LL tumor environment, which was then counteracted following inhibition of COX-2 (9). This augmented expression of Nr4a factors by COX-2/PGE2 axis may contribute to the different characteristics between the lymphoid and tumor Treg cells discussed above.

Collectively, although the behavior of Nr4a factors is similar between lymphoid and tumor Treg cells, some differences do exist, which seem to stem from differences in their environments, including changes in the metabolite and cytokine milieu surrounding these cells in these two tissues. However, further experimental validation is required.

## Concluding Remarks

Treg cells are an attractive target for therapeutic reactivation of the the antitumor immune response in various cancers. However, the high degree of similarity between lymphoid and tumor Treg cells makes the targeted suppression of tumor Treg cells difficult, often causing unwanted side effects that negatively impact the lymphoid Treg cells. Thus, studies that clarify the differences between these two cell populations may have significant clinical value. This review described the differences in the behaviors of the Nr4a factors in lymphoid and tumor Treg cells, while touching on the many overlaps between these cells. These evaluations identify COX-2/PGE2 as an attractive target for therapeutic intervention as this axis is exclusively augmented in specific tumor tissues, with the PGE2-rich TME upregulating Nr4a factors and increased stabilization of tumor Treg cells. This means that molecules that inhibit this pathway are expected to reactivate antitumor immunity as seen in the animal model, with little to no effect on the lymphoid Treg cells. Molecules that specifically inhibit Nr4a1 and Nr4a2, but not Nr4a3, are also candidates for tumor Treg-specific inhibitors, as Foxp3^Cre^Nr4a-DcKO mice do not develop autoimmunity but the mice do develop augmented antitumor immunity.

Another caveat preventing effective immune response reactivation *via* Treg cell inhibition is the fundamental similarity between Treg cells and conventional T cells, including cytotoxic T cells that mediate antitumor immunity. This is exemplified by the fact that many chemotherapeutic agents that inhibit Treg cell proliferation, including cyclophosphamide and cyclosporine A, also exhibit significant toxicity against conventional T cells as well (44, 45). PD-1 blockade has also been reported to not only activate Teff cells, but also augment the immune suppressive activity of Treg cells, leading to the progression of gastric cancer (46). Given this, Nr4a factors surpass many other candidate targets, as they show a repressive effects on conventional T cells by inducing their dysfunction (24, 25).

Taken together, current data suggest that Nr4a factors are crucial regulators of Treg cells and are attractive targets for novel cancer immunotherapy. Thus, we suggest that a comprehensive comparison of Nr4a factors between lymphoid Treg and tumor Treg cells, including their cooperating partners and target genes, should help to identify additional differences between these settings and thus novel targets for the specific disruption of tumor Treg cells.

## Author Contributions

TS wrote the manuscript. The author confirms being the sole contributor of this work and has approved it for publication.

## Funding

This work was supported by JSPS KAKENHI Grant-in-Aid for Scientific Research (B) 21H02756, for Scientific Research on Innovative Areas 19H04822 and 20H04958, for Challenging Exploratory Research 21K19393, the Takeda Science Foundation, and Grant for National Center for Global Health and Medicine (20A1005).

## Conflict of Interest

The author declares that the research was conducted in the absence of any commercial or financial relationships that could be construed as a potential conflict of interest.

## Publisher’s Note

All claims expressed in this article are solely those of the authors and do not necessarily represent those of their affiliated organizations, or those of the publisher, the editors and the reviewers. Any product that may be evaluated in this article, or claim that may be made by its manufacturer, is not guaranteed or endorsed by the publisher.
